# The Global Health Security Index: Another Look

**DOI:** 10.3389/fepid.2022.846260

**Published:** 2022-06-13

**Authors:** Peter G. Goldschmidt

**Affiliations:** World Development Group, Inc., Bethesda, MD, United States

**Keywords:** global, pandemic, performance, policy, preparedness, response, validity, COVID-19

## Abstract

The Global Health Security Index (GHSI) was published in October 2019 (after more than 2 years in preparation); at about the same time that the COVID-19 pandemic (COVID) started. The GHSI was intended to score countries' pandemic preparedness. Within months of the start of the pandemic, articles began to be published that claimed to assess the GHSI's validity. They correlated GHSI scores with countries' COVID per capita death rates. They showed that the better prepared a country, the higher the death rate: a result that was counter to what would have been expected. This article takes another look at the GHSI by exploring the relationship in major European Union countries plus the United Kingdom. The analysis reported here confirms that early on the higher the GHSI score, the higher the COVID per capita death rate (*r* = 0.52, *P* < 0.05). But, by the end of 2020, there was no correlation. By July 2021, the correlation was in the expected direction: the higher the GHSI score, the lower the COVID per capita death rate (*r* = −0.55, *P* < 0.05); ditto case fatality rate (*r* = −0.74, *P* < 0.01). Further, the GHSI was better correlated with excess mortality, the best measure of pandemic impact (*r* = −0.69, *P* < 0.01). However, per capita GDP was as good a predictor of excess mortality (*r* = −0.71, *P* < 0.01) and the Health System Performance Index of case fatality rate (*r* = −0.71; *P* < 0.01). By the end of 2021, the correlation between GHSI scores and COVID per capita death rates had strengthened (*r* = −0.71; *P* < 0.01). This exploratory analysis is not intended to produce generalizable conclusions about the effectiveness of countries' COVID pandemic response management, which continues to evolve and hence can only be properly assessed after the pandemic has ended. Nevertheless, the following conclusions would seem to be warranted: 1) there seems to have been a rush to judge, or, at least, to publish, and 2) the validity of any forward looking pandemic preparedness score depends not only on being able to assess countries' capabilities but also being able to forecast what governments will (and will not) do in any given situation, a seemingly quixotic quest.

## Introduction

### Government Failings

COVID-19 surprised the world. Governments were unprepared to respond to the pandemic, despite (1) decades of warnings that such a pandemic was inevitable (1) and (2) numerous articles about lessons learned from past pandemics (2, 3). Most governments lost valuable time in mobilizing a realistic response and unleashed a potentially avoidable social catastrophe (4). Leaders in many countries initially underestimated the pandemic's severity; in others, simply resisted introducing extraordinary measures that might frighten people or cause economic disruption (5). They not only failed to base policy on sound science but also acted contrary to what was needed, which further reduced already low levels of trust in institutions, even as the accompanying infodemic further eroded trust in public health authorities (6).

### Pandemic Preparedness

The Global Health Security Index (GHSI) was published in October 2019 (7), at about the time that the COVID-19 pathogen started to circulate among human populations. Index scores were intended to reflect a country's preparedness to respond to a pandemic. Rarely does the opportunity arise to evaluate the validity of such theoretical scores. Unfortunately, the COVID-19 pandemic (COVID) provided one. The goal of pandemic response management (PRM) is to minimize deaths, while simultaneously minimizing socioeconomic damage resulting from PRM interventions, policies and strategies (PASS) (8). During the early months of the pandemic several authors published analyses of the relationship between a country's GHSI score and its COVID per capita death rate. As Table 1 shows, these early assessments of the validity of the GHSI reported an unexpected correlation which they could not explain: the higher the GHSI score the higher the COVID per capita death rate (9–14). Further, several of these authors noted that certain countries had a mediocre or low GHSI score but had done well in managing COVID. Some extolled Vietnam's performance (9, 11, 14); noting that the country had recent experience in managing epidemics (9).

**Table 1:** Early conclusions of the relationship between GHSI score and pandemic performance [a]: The higher the GHSI score, the higher the selected parameter (i.e., opposite to the expected direction).

**Date of analysis**	**Countries included**	**Selected parameter**	**Method**	**Strength**	**Reference**
Mar 2020	[b]	Deaths/million [c]	Spearman correlation	0.31	(9)
Apr 2020	100 with data	Deaths/ million/day	Log trans plot	–	(10)
May 2020	36 OECD	Performance composite	Spearman correlation	0.41 *P* <0.05	(11)
Jun 2020	29 selected	Deaths/ million	Rank order difference	–	(12)
Aug 2020	167 with deaths	Deaths/ million	Correlation	0.41 *P* <0.05	(13)
Oct 2020	[b]	Deaths/ million	Correlation	0.35 *P* <0.01	(14)

*[a] Limited to journal publications. The methods section is often unclear; results section, sketchy*.

*[b] Not stated; based on graph points, presumed to be all with a GHSI score (195) and death data (unstated number)*.

*[c] Compared to the mean value of GHSI category 2; grouped into three classes. Data were also analyzed for July 2020 but only the stated strength of association was given*.

### Global Health Security Index

In the aftermath of the 2014/15 West Africa Ebola epidemic, several preparedness projects were undertaken. They included the Global Health Security Index (GHSI): the first comprehensive assessment of global health security capabilities to respond to a pandemic or other public health emergency. The GHSI covers six categories: (1) prevention, (2) detection and reporting, (3) rapid response, (4) health system, (5) compliance with international norms, and (6) risk environment (7). Capabilities were assessed for the 195 countries that are State Parties to the International Health Regulations (IHR), using publicly-available sources. The GHSI provides a global index score ranging from zero (least prepared) to 100 (most prepared). The USA was determined to be most prepared (83.5) and the UK next best (77.9); least prepared, Equatorial Guinea (16.2) (7). The GHSI is a broader assessment than the earlier World Health Organization (WHO) Joint External Evaluation (which was developed to provide a more transparent independent and objective assessment of a country's ability to comply with IHR requirements) (15).

### WHO Health System Performance Index

In June 2000, the WHO published the better known Health System Performance Index (HSPI): the first ever analysis of its 191 members states' health systems (16). France came out top. The UK ranked 18th and the USA ranked 37th (despite spending by far the most money per capita) (17). The assessment system was based on five indicators: (1) overall level of population health, (2) health inequalities (or disparities) within the population, (3) overall level of health system responsiveness (a combination of patient satisfaction and how well the system acts), (4) distribution of responsiveness within the population (how well people of varying economic status find that they are served by the health system), and (5) the distribution of the health system's financial burden within the population (who pays the costs) (16). The intention was to update rankings at regular intervals (18); none seems to have been forthcoming. Subsequent research published a year after the WHO rankings found little relationship between them and citizens' perceptions in 17 industrialized countries (19). In 2019, the WHO published a report on universal health care service coverage, measuring progress from 2000 to 2017 on UN sustainable development goal indicator 3.8.1; also separately indicator 3.8.2 (health expenditures in relation to household budget) (20). Analyses of similar indexes have also been reported (21, 22).

### Purpose/Scope of This Article

This paper (1) revisits the relationship between GHSI scores and COVID death rates, and also case fatality rates (CFR), (2) examines the relationship between WHO HSPI scores and COVID CFR and between gross domestic product and excess mortality, and (3) discusses forward-looking index scores in the context of global pandemic preparedness. The methods section describes the basis for this exploratory analysis and choice of PRM performance measures. The discussion section includes an alternative approach to assessing and evaluating pandemic preparedness.

## Methods

### Correlations

This exploratory study examines the association between GHSI and HSPI index scores (7, 23) and corresponding pandemic outcomes, COVID case fatality rates and per capita death rates (24) and also excess mortality (25) based on the Pearson Product Moment Correlation applied to published data for pertinent points in time. Certain additional associations were similarly explored, including that between 2019 per capita gross domestic product (GDP), calculated in terms of purchasing power parity (PPP) (26) and excess death rate. The population of 20 countries chosen for this study consisted of the 19 major European Union (EU) countries (those with a population of at least 5 million people) plus the United Kingdom (UK), which was effectively a member until the end of 2020. The EU countries were Austria, Belgium, Bulgaria, Czech Republic, Denmark, Finland, France, Germany, Greece, Hungary, Italy, Netherlands, Poland, Portugal, Romania, Slovakia, Spain, and Sweden. These countries were chosen based on (1) their geographic contiguity (so that the dynamics of pandemic spread would be less of a factor than comparing countries on widely separated continents), (2) advanced development (so that data used to calculate index scores and COVID experience can be considered to be at least somewhat reliable), (3) roughly comparable population age-sex structures (because COVID death rates vary by these parameters), and (4) sufficient range of index scores. The GHSI scores ranged from 77.9 for the UK to 45.6 for Bulgaria; HSPI scores, from 0.994 for France to 0.639 for Bulgaria (on a zero to one scale). Of particular interest was the dynamic relationship of the GHSI score to COVID per capita death rates as the pandemic unfolded. The following 3 points in time were chosen: (1) July 2020, (2) the end of 2020, and (3) July 2021. For context and comparison purposes, for July 2021, additional relationships were explored (matching index to pertinent outcome): (1) the GHSI was correlated with excess mortality rate and COVID case fatality rate, (2) the HSPI score with case fatality rate, and (3) GDP/PPP with excess mortality rate. Pearson correlations were performed using an online calculator. Data used in these analyses were from cited sources. All measurements are subject to the quality of data resulting from mechanisms used to make them. Comparing measurements among different countries assumes implicitly that underling data are similarly fit-for-purpose. The remainder of this section describes the choice of COVID PRM measures used in this exploratory analysis. This article is not intended to be a primer on epidemiology; further explanations and discussion of limitations of measurements, especially when comparing countries, is beyond its scope.

### Selection of Pandemic Response Performance Measure

#### Immediate Impact: Death

Various measures have been suggested or used for assessing PRM performance, including excess mortality, death rates, case rates, case fatality rates (all of which are used in this paper for purposes of illustration), and R0 (the basic reproduction number, which indicates a pathogen's contagiousness at the start of a pandemic when everyone in a population is susceptible to the disease). R0 is not an appropriate PRM performance measure (27). Death is the most immediate impact of a pandemic on population health. Total population health impact (1) may only be revealed long after a pandemic has ended (as exemplified by long- COVID and other potential long-term health consequences among survivors) and (2) may never be able to be assessed reliably due to measurement limitations. Pandemic deaths include those caused (1) directly by the pathogen and (2) indirectly due to a pandemic's socioeconomic impacts, including from PRM. For COVID-19, pertinent data are limited, unreliable, not comparable across countries, and, in some cases may simply be fake (28, 29).

#### Excess Mortality

Excess mortality was first used more than 350 years ago in connection with the London plague (30), and is still considered to be the best measure of a pandemic's impact (25, 31). It involves subtracting expected mortality (e.g., the average experienced in the 5 years before a pandemic) from mortality observed during the pandemic. Excess mortality has several advantages, including (1) there is no need to determine who died due to COVID (which may be difficult to determine and ascertainment may vary by jurisdiction) and (2) it accounts for collateral deaths (e.g., when a patient dies of an unrelated cause because all hospital beds are occupied by pandemic victims or when a quarantined person commits suicide). Excess mortality can be negative, i.e., fewer people than expected die during the pandemic period, possibly due to PRM (as was experienced in Denmark). Excess mortality data are not readily available presently. For countries included in the analysis, such data became available in time to be included as a mid-2021 point of comparison (25).

#### Per Capita Death Rate

Per capita death rates are currently the preferred PRM performance measure (even if less than perfect) (32), in part because they are readily available on a daily basis and are widely tracked. The per capita death rate is the proportion of a population that died of COVID in a given period (often expressed as deaths per 100,000 population). Ascertaining how many people actually contracted COVID is difficult (1) because some cases exhibit only mild symptoms or are asymptomatic and (2) variations in testing rates and accuracy produces variations in case counts. Factors complicating comparative measurements include (1) a population's age-sex structure and (2) in some cases, the prevalence of pre-existing conditions, such as obesity and diabetes. COVID-19 death rates vary based on many factors, and are acknowledged to increase with age and in the presence of certain conditions. Thus, one can expect a country's population pyramid and health to affect its death rate. For the countries considered in this paper, this is less of an issue than it would be in comparing PRM performance across all countries of the world. Further, COVID-19 seems to have simply amplified baseline mortality risk to approximately the same relative degree for most population subgroups, at least in the UK (33). The USA has a COVID-19 death rate that is more than 80 times that of Taiwan (24). In the USA, the highest age-adjusted COVID-19 death rate of any of its 50 states is 5.4 times that of the lowest state. Further, adjusting for age, did little to change states' rate or ranking (34). Little is known about the factors that explain the great variation observed in COVID-19 death rates. Scientific research on reasons death rates vary tremendously is limited, and what research exists is mostly inadequate and/or inconclusive. Regardless, one could argue that when responding to a pandemic, a properly prepared country should be able to accommodate the specific requirements of its population.

#### Case Rate

The case rate (CR) is the number of new cases of an infection that arise in a population during a given period divided by the size of that population. The CR is often expressed as the number of cases per 100,00 population. Case rates are unreliable and some authors have explicitly ruled them out as an appropriate PRM performance measure (32, 35), in part, because a country's performance depends on its health system capabilities and the effectiveness with which they are deployed; hence their influence on death rates. Moreover, for COVID, most cross-country variation in cumulative infection rates could not be explained (36).

#### Case Fatality Rate

The case fatality rate (CFR) is the percentage of people with a confirmed COVID diagnosis who died of the disease. The validity of the result depends on various decisions, which despite any common rules may nevertheless vary by jurisdiction. They include (1) health system capacity and care seeking behavior, (2) diagnostic accuracy, and (3) consistency in determining cause of death. Limitations of the COVID CFR are similar to those for the per capita death rate. Further, one could consider that the CFR is most sensitive to the degree of development of a country's health system; it is not an appropriate measure of a country's PRM performance.

## Results

Results are presented in 2 parts. The first part reports the change in correlation between the GHSI score and COVID per capita death rate; the second elaborates on the situation as of July 2021. As Table 2 shows, early on in the COVID pandemic, at the end of July 2020, there was a moderate correlation between the GHSI score and COVID per capita death rate in the 20 countries included in the analysis, but in an unexpected direction: the more prepared a country to respond to a pandemic, the higher its death rate (*r* = 0.52, *P* < 0.05). By the end of 2020, there was no correlation and by the end of July 2021 there was a moderate correlation in the expected direction: the higher the GHSI score the lower the death rate (*r* = −0.55, *P* < 0.05). Of note, four of the five countries with the lowest per capita death rate in July 2020 by July 2021 were now among the top 5 (Bulgaria, Czech Republic, Hungary, and Slovakia). In July 2021, there was an even stronger correlation between GHSI score and excess mortality (*r* = −0.69; *P* < 0.01). Further, the correlation between per capita GDP/PPP and excess mortality was stronger still (*r* = −0.71; *P* < 0.01). The correlation between GHSI score and July 2021 case fatality rate was similarly strong (*r* = −0.74; *P* < 0.01), as was that between the HSPI score and CFR (*r* = −0.71; *P* < 0.01). In other words, in July 2021, for the 20 countries included in the analysis, correlations were in the expected direction—and reassuringly strong. By the end of 2021, the association between GHSI score and COVID per capita death rate had strengthened further, to −0.71 (*P* < 0.01).

**Table 2:** Relationship of selected parameters to pandemic outcomes in major EU countries plus UK—Pearson product moment correlation: negative sign indicates the higher the GHSI or HSPI score or GDP, the lower the death or case fatality rate; positive sign, vice versa.

**Relationship**	**Mid-2020**	**End-2020**	**Mid-2021**	**End-2021**
**Principal PRM**				
**performance measure**				
**GHSI score vs**.				
- Covid per capita death rate	+0.52 *P* <0.05	None	−0.55 *P* <0.05	−0.71 *P* <0.01
**For context and comparison**				
- Excess mortality rate			−0.69 *P* <0.01	
- Case fatality rate			−0.74 *P* <0.01	
**Additional correlations**				
**WHO HSPI score vs**.				
- Case fatality rate			−0.71 *P* <0.01	
**2019 per capita GDP vs**.				
- Excess mortality rate			−0.71 *P* <0.01	

## Discussion

### Early Reports

During the first 6 months of the pandemic more than 23,500 articles about COVID were published (37). The WHO COVID database now likely contains more than 250,000 articles (38). After more than 2 years in preparation, the GHSI was published in October 2019, at the same time as the COVID-19 pathogen started to circulated among human populations (7). The GHSI project's goal was to assess countries' preparedness to respond to pandemics. How well did the GHSI predict COVID PRM performance? Several published studies, using similar methodologies, but different populations of countries, reported that the higher a country's GHSI score the higher its COVID per capita death rate. Study authors and commentators searched for an explanation as to why the most well-prepared countries did worse (9–14, 39, 40).

### Searching for Explanations

Some of these authors pointed to the inability to capture the competence of state institutions (39); others explained the disconnect between countries' GHSI score and death rate in terms of failure to anticipate or to account sufficiently for leadership, as evident in GHSI high-scoring countries, such as the USA (40). Some authors extolled selected countries stellar performance, including that of Vietnam (9, 14, 39). Its early PRM performance was described as enviable, despite its comparatively low GHSI score (49.1) (14); perhaps because of its prior experience in 2003 with SARS (a prior coronavirus pandemic) (41) or more recent epidemics (9). The country reported its first COVID case on July 1, 2020. The daily COVID case count remained mostly in single digits until April 2021, when it began to surge. By September 2021 cases exceeded 13,000 per day. Vietnam instituted one of the world's strictest lockdowns (in Ho Chi Min City) (42); convicted quarantine rule breakers were sentenced to up to 5 years in jail (43). When the COVID delta variant arrived, it simply overwhelmed the country's ongoing PRM efforts and capabilities (44). Later, the omicron variant overwhelmed almost all countries' pandemic suppression capabilities. China managed to continue containing the pandemic with its strict dynamic zero- COVID strategy (45). The quest to find the variable that explains countries' differing COVID per capita death rates continues, with inequality being the latest implicated factor (46). Reality is likely much more complicated.

### Another Look

Results of the analysis reported here confirmed those of cited study authors, as of July 2020. But, for the 20 countries analyzed, by the end of 2020, there was no relationship between the GHSI score and reported COVID per capita death rate. By July 2021, the correlation was in the expected direction: the higher the GHSI, the lower the COVID per capita death rate. Moreover, by the end of 2021, this expected correlation had further strengthened. Similarly, as of July 2021, the WHO HSPI score (already more than 20 years old) was about as good a predictor of COVID CFR as the GHSI score.

### Overconfidence

The greatest weakness in PRM is overconfidence. The most important lesson is what COVID revealed about governments (47). From the start, all but a few governments failed abysmally in responding to COVID, as a high-powered independent panel has already concluded (4). For a multiplicity of different reasons, countries failed somewhere along the pandemic timeline; some sooner than others; some more so than others. Premature evaluations, such as early correlations of GHSI scores with death rates, can easily be overcome by events. Cited study authors appear to have rushed to judgment, as the pandemic was unfolding, so that their analyses could not account for (1) the (often complicated and unpredictable) dynamics of pandemic spread and (2) countries true PRM capabilities (and good luck). COVID did not strike everywhere at the same time with the same force; the coronavirus also evolved. What does that mean in this context? Consider the following analogy. A castle may appear to be well-defended and its defenders in a strong position, if invaders launch only intermittent, half-hearted attacks. When they ramp up the frequency and force of their attacks, castle defenders may be quickly overrun, revealing their true strength. Invaders may first set their sights on castles in a particular region and then move on to others. At any given moment, overrun castles may be seen to have been weakly-defended while others still appear to be strongly-defended. As invaders surge across the landscape, overrunning castles as they go, all castles may eventually be seen to have been truly weakly defended.

### Study Limitations

The limitations of this study include: (1) the PRM experience of only a single sample of countries was analyzed and (2) the reported correlations may evolve further before the pandemic ends. Its strengths include: (1) the countries analyzed are in a the same geographic region and are among the world's most advanced economies and (2) 24 months has elapsed since the pandemic struck and it is considered to be winding down as these countries are already transitioning to the new normal (while the pandemic still continues to unfold worldwide).

### Quixotic Quest

Countries with higher GHSI score should achieve better PRM performance (manifest when correlating GHSI scores with COVID per capita death rates). And so it is with the GHSI, at least for the countries analyzed here as of July 2021 and reaffirmed as of the end of 2021. However, per capita GDP/PPP is as good a predictor of excess mortality. Capabilities (encapsulated in the GHSI and HSPI) may simply be related to the strength of a country's economy: wealthier countries can be expected to have (or at least afford) better health systems (and to be better prepared to deal with public health emergencies). As COVID demonstrated, no amount of pandemic preparedness can overcome poor or failed leadership and/or erroneous policies. Since PRM performance depends not only on available capabilities but also on leadership and the effective implementation of policies, the GHSI must predict years in advance what governments will (and will not do) in any given situation. Such predictions are difficult and controversial, especially when large countries may have semi-autonomous regional governments. Hence, constructing a predictive pandemic preparedness index is akin to tilting at windmills; an excise in futility if not a fool's errand.

### Rush to Publish

Within the first several months of COVID, there seems to have been a rush to evaluate the GHSI as a predictor of countries' pandemic response management—or, at least, a rush to publish. The effectiveness of countries PRM can only be properly evaluated after a pandemic ends; impacts, only decades later. This article reports results of an exploration of the validity of the GHSI based on COVID experience in the first 24 months of the pandemic in 19 major EU countries plus the UK. This exploratory study demonstrates that (1) premature evaluation results can be overcome by events and (2) the pursuit of an index of pandemic preparedness is a quixotic quest. Going forward a more appropriate approach is needed (1) to assess pandemic preparedness and (2) to evaluate PRM performance.

### Alternative Approach

How can countries' PRM preparedness and performance be realistically assessed, especially when (1) PRM involve country-specific choices and (2) they have different contextual backgrounds, infrastructures, and resources? The most appropriate approach is to assess PRM based on (1) achievable preparedness (manifest in a pandemic playbook) and (2) achievable performance achieved (when struck by a pandemic). Proper pandemic preparedness requires maintaining a realistic pandemic playbook (PPB) that a country is ready to implement when a pandemic strikes. A PPB system is necessary to maintain such a playbook (48). Over time, a country could adjust its PPB not only based on results of periodically exercising it but also in step with improvements in its contextual background, infrastructures, health system, etc. After a pandemic (or epidemic) evaluation of a country's PRM performance should include 1) an assessment of achievable performance achieved (and reasons for any over- or under-achievement) and 2) priorities for upgrading the contextual background, infrastructures, health system, etc and improving the PPB system, PPB PASS, and implementation plans and mechanisms (including enabling public health legislation). The task ahead is (1) to develop an international management system standard to guide countries in maintaining a realistic PPB and (2) to establish a mechanism not only to certify countries compliance with process requirements but also to assess the quality of their implementation. On this basis, it would be possible to assess (1) how well-prepared a country is to manage a pandemic within its circumstances and (2) after a pandemic, how well it actually managed it. International technical assistance and, where applicable aid, (1) could usefully support countries in developing and operating a PPB system and (2) could assist countries with low preparedness and/or poor performance to improve, because in a pandemic no-one is safe until everyone is safe.

### Going Forward

In order to prepare for the next pandemic, countries should establish a PPB system. The existence of a realistic, up-to-date PPB may be sufficient evidence that the country (1) could actually implement its PASS when the next pandemic strikes and (2) could adjust its PRM based on the evolving situation on the ground. Building an appropriate pandemic playbook system, rectifying infrastructure deficiencies revealed by COVID, and accommodating the new normal contextual background is likely to be a many-year project—all the more reason to start now, to be better prepared whenever the next global pandemic strikes.

## Data Availability Statement

Publicly available datasets were analyzed in this study. This data can be found here: https://coronavirus.jhu.edu/data/mortality; https://www.ghsindex.org/wp-content/uploads/2019/10/2019-Global-Health-Security-Index.pdf; https://www.who.int/healthinfo/paper30.pdf; https://www.medrxiv.org/content/10.1101/2021.01.27.21250604v3; https://data.worldbank.org/indicator/NY.GDP.MKTP.PP.CD (as referenced in the article).

## Author Contributions

The author confirms being the sole contributor of this work and has approved it for publication.

## Conflict of Interest

PG is the Founder/President of the World Development Group, Inc., a consultancy.

## Publisher's Note

All claims expressed in this article are solely those of the authors and do not necessarily represent those of their affiliated organizations, or those of the publisher, the editors and the reviewers. Any product that may be evaluated in this article, or claim that may be made by its manufacturer, is not guaranteed or endorsed by the publisher.
